# Cognitive decline and mortality among community-dwelling Chinese older people

**DOI:** 10.1186/s12916-019-1295-8

**Published:** 2019-03-15

**Authors:** Xiaozhen Lv, Wenyuan Li, Yuan Ma, Huashuai Chen, Yi Zeng, Xin Yu, Albert Hofman, Huali Wang

**Affiliations:** 1 Dementia Care and Research Center, Clinical Research Division, Peking University Institute of Mental Health (Sixth Hospital), No. 51 Huayuanbei Road, Haidian District, Beijing, 100191 China; 2 Beijing Dementia Key Lab, National Clinical Research Center for Mental Disorders, Key Laboratory of Mental Health, Ministry of Health (Peking University), Beijing, China; 3 Department of Epidemiology, Harvard T.H. Chan School of Public Health, 677 Huntington Avenue, Boston, MA 02115 USA; 4 Center for the Study of Aging and Human Development, Medical School of Duke University, Box 3003, Room 1506 Busse Building, Blue Zone, Duke South, Durham, NC 27710 USA; 5 International Trade Department, Business School of Xiangtan University, Xiangtan, Hunan China; 6 Center for Healthy Aging and Development Studies, National School of Development, Raissun Institute for Advanced Studies, Peking University, No. 5 Yiheyuan Road, Haidian District, Beijing, 100875 China

**Keywords:** Cognitive decline, Mortality, Cohort, Community-based, Chinese older people

## Abstract

**Background:**

Whether cognitive decline is related to a higher risk of death independent of the initial cognitive function is inconclusive. Evidence of the association between cognitive decline and mortality among Chinese older people is limited. We aimed to examine whether cognitive decline, assessed by the rate of decrease in the Mini-Mental State Examination (MMSE) score, was associated with mortality independent of initial cognitive function (baseline MMSE score) among Chinese older people.

**Methods:**

We established two successive and non-overlapping cohorts of older adults nested within the Chinese Longitudinal Healthy Longevity Survey (CLHLS), an ongoing, open, community-based cohort survey conducted every 2–3 years. Cognitive function was measured using the Chinese version of the MMSE. A total of 11,732 older adults who completed two consecutive cognitive function examinations were included and followed for 3 years. A Cox proportional hazards model was used to examine the association of cognitive decline with mortality after adjusting for sociodemographic characteristics, health behaviours, comorbidities and initial cognitive function.

**Results:**

The mean age was 82.5 years old, and 44.9% (5264/11732) of participants were men. After adjusting for baseline MMSE scores and other covariates, the rate of change in MMSE scores over 3 years was monotonically and positively associated with subsequent 3-year mortality. Compared to those with stable cognitive function, participants with rapid cognitive decline (decline faster than average, a reduction of MMSE scores > 1.62 points/year) had a 75% higher risk of death (hazard ratio = 1.75, 95% confidence interval 1.57–1.95). The association between cognitive decline and mortality was stronger among relatively younger Chinese older people (aged 65–79 years versus 80 years and over) and those with normal cognitive function at baseline (MMSE scores ≥ 24 versus < 24 points), respectively, but did not differ by cohort and sex.

**Conclusion:**

Faster cognitive decline was associated with higher mortality independent of initial cognitive function, especially among those aged 65–79 years and those with normal cognitive function at baseline. The association was consistent across two successive cohorts. Our findings indicate the practical significance of monitoring cognitive change in older adults.

## Background

With the global population ageing, dementia is a growing public health concern [1]. Preservation of cognitive function is a central component of successful ageing [2]. Numerous studies have shown that lower cognitive function is associated with an increased risk of death [3–10].

It was suggested that cognitive function generally declines with age among older adults [11], and the rate of cognitive decline (i.e. reduction in cognitive function) is related to initial cognitive function [12]. However, it is inconclusive whether cognitive decline is independently associated with mortality. Most previous studies found that faster cognitive decline was associated with an increased risk of death after adjusting for initial cognitive function [5, 13–18]. However, Bruce et al. reported that the association between cognitive decline and mortality attenuated to null when adjusted for baseline cognitive function [19]. Bosworth et al. found that time to death was not highly related to changes in intellectual performance [20]. Hassing et al. found that the decedents did not decline more rapidly in all 11 cognitive measurements across a 4-year period than the survivors [8]. Discrepancies may partly be explained by relatively small sample sizes [8, 14, 16–18, 20] or differences in age distributions in previous studies [15, 19, 20]. China has the largest ageing population and is one of the fastest ageing countries in the world [21]. To our knowledge, the association between cognitive decline and mortality has not been thoroughly evaluated among a national cohort of Chinese older people.

In addition, it has been reported that the cognitive function of older adults might change at different times owing to changes in education, comorbidities and social progress [22, 23]. The strength of the association between cognitive function and mortality might accordingly change over time [22, 24]. However, whether the association of cognitive decline with mortality differs at different times is unknown.

The Chinese Longitudinal Healthy Longevity Survey (CLHLS) includes a dynamic, prospective and national cohort of community-dwelling Chinese older people. In this study, we investigated whether cognitive function change over 3 years was associated with subsequent 3-year mortality after adjusting for baseline cognitive function and determined whether the association differed in two large, successive and non-overlapping cohorts within the CLHLS.

## Methods

### Study design and participants

The CLHLS is an ongoing, prospective cohort study of community-dwelling Chinese older people. Details can be found elsewhere [25–27]. Briefly, the CLHLS is a nationwide survey covering approximately 85% of China’s population and provides representative data to investigate determinants of longevity. It began in 1998, and examinations are carried out every 2–3 years. To reduce the attrition due to death and loss to follow-up, new participants are enrolled during the follow-up. The surveys are administered in participants’ homes by trained interviewers with a structured questionnaire. Proxy respondents, usually a spouse or other close family members, are interviewed when the participants are unable to answer questions, but questions regarding cognitive function and mood are answered by participants themselves.

**Fig. 1:**
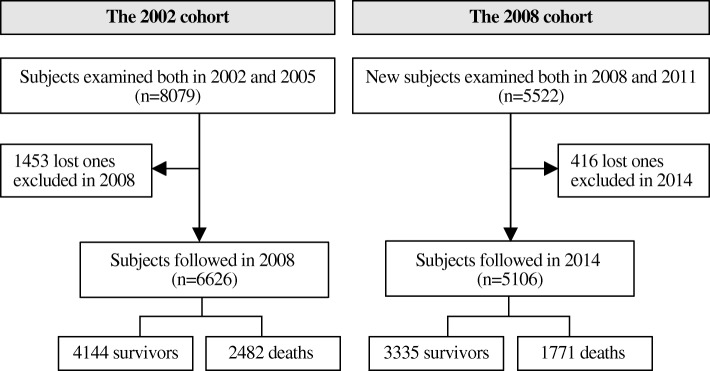
Flow chart of the study population

The current study was based on two successive and non-overlapping cohorts (the 2002 and 2008 cohorts) within the CLHLS. Three surveys were performed in each cohort (2002, 2005 and 2008 in the 2002 cohort and 2008, 2011 and 2014 in the 2008 cohort). We included 13,601 participants who were free of dementia at baseline (the first survey) and completed cognitive function assessments during both the first and second surveys. We then excluded 1869 participants who were lost to follow-up before the third survey. A total of 11,732 participants were included as the study population, with 6626 from the 2002 cohort and 5106 from the 2008 cohort (Fig. 1).

The CLHLS study was approved by the Research Ethics Committee of Peking University (IRB00001052-13074), and all participants or their proxy respondents provided written informed consent.

### Outcome

The participants’ survival status and date of death were collected through interviews with close family members during the third survey in each cohort.

### Exposure

Cognitive function was measured by the Chinese version of the Mini-Mental State Examination (MMSE) during each survey. The validity and reliability of the Chinese MMSE has been verified [9, 25, 26]. Based on the literature [28], we treated responses of “unable to answer” as “wrong”. The MMSE score ranged from 0 to 30, and a higher score indicated better cognitive function.

Cognitive decline was assessed using the rate of change in MMSE score, which was calculated as the difference between the MMSE score at baseline and the second survey divided by the number of years between the two examinations ((MMSE score at baseline − MMSE score at the second survey)/the interval between two examinations, years). We then categorized the rate of change in the MMSE score into four groups: cognitive improvement (a rate of change in MMSE score less than zero), stable cognitive function (a rate of change in MMSE score equal to zero), slow decline (a rate of change in MMSE scores greater than zero but equal to or less than the median of those showing decline [rate of change in MMSE score greater than zero]) and rapid decline (a rate of change in MMSE score greater than the median of those showing decline).

### Covariates

We adjusted sociodemographic characteristics, health behaviours and health status at baseline in the model. These covariates were selected a priori as potential confounders based on the literature [3, 9, 10, 15, 18, 24, 29]. The sociodemographic characteristics were age (continuous), sex, education (no schooling/some schooling), main occupation (white-collar/other), residence (urban/rural), marital status (married/other) and co-residence with family members (yes/no). Health behaviours were smoking status (never/past/current), current alcohol intake (yes/no) and regular exercise at present (yes/no). Health status was common chronic diseases (self-reported hypertension, diabetes, heart disease, cerebrovascular disease and respiratory disease), body mass index (BMI, underweight (< 18.5)/normal (18.5–23.9)/overweight (24–27.9)/obese (≥ 28) [30]) and symptoms of psychological distress (measured based on loneliness, usefulness and fearfulness [31], categorized into four groups according to the quartile of the total score). In addition, a cohort identifier was established to indicate which cohort the participants were from.

### Statistical analyses

Cox proportional hazards models were constructed to determine the association of cognitive decline with mortality. The follow-up started from the second survey and ended on the date of death or the end of the study (the third survey), whichever was earlier. The endpoint events were defined as death or the end of the study, whichever came first. A small percentage of the data for most covariates were missing (< 3%), with the exception of symptoms of psychological distress (7.8%). For these missing values, we used missing indicators. We ascertained that the proportional hazard assumption had not been violated.

Cognitive decline was modelled as both a continuous variable (rate of change in MMSE score) and a categorical variable (improvement, stable, slow decline and rapid decline). Data are reported as adjusted hazard ratios (HRs) and 95% confidence intervals (CIs). The adjustment was accomplished via three stages: (1) we adjusted for age and sex; (2) we added the cohort identifier, education, occupation, type of residence, marital status, co-residence, smoking, alcohol intake, and exercising, hypertension, diabetes, heart disease, cerebrovascular disease, respiratory disease, BMI and symptoms of psychological distress; and (3) we additionally adjusted for baseline MMSE score (the fully adjusted model).

To examine the shape of the association between the rate of change in MMSE score and mortality, we conducted a restricted cubic spline analysis. We examined whether the association of cognitive decline with mortality differed by cohort, sex, age (65–79 versus ≥ 80 years old) and baseline cognitive function (MMSE score ≥ 24 versus < 24 points) by separately adding an interaction term to the fully adjusted model.

To address the issue of loss to follow-up, we conducted a sensitivity analysis by considering the losses censored at the end of the study. We also performed a sensitivity analysis by excluding deaths that occurred in the first year of the follow-up to account for the possibility that a pre-mortality drop in cognitive function and/or disease status in the last year of life could have influenced our results.

A two-tailed *P* value < 0.05 was used to determine statistical significance. All analyses were performed with SPSS 20.0 for Windows (IBM SPSS Inc., Chicago, IL, USA) except for the restricted cubic spline analysis, which was performed using R version 3.4.2 (R Foundation for Statistical Computing).

## Results

### Participant characteristics

The mean (standard deviation) age was 82.5 (11.3) years old at baseline, and 44.9% of participants (5264/11732) were men. Approximately 60% of the individuals were illiterate. There were 29.2% participants with cognitive impairment at baseline and 37.4% with cognitive impairment at the second survey. The median (25th, 75th percentile) of the rate of change in MMSE score was 0.0 (− 1.6, 0.6) points per year. At a median of 2.8 years of follow-up, 4253 (36.3%) deaths were identified. The characteristics of participants by cohort are shown in Table 1.

**Table 1:** Characteristics (data are expressed as counts (percentages) except when specified otherwise) of 11,732 community-dwelling Chinese older people at baseline by cohort

	2002 cohort (*n* = 6626)	2008 cohort (*n* = 5106)	Total (*n* = 11,732)
Age, years, mean (standard deviation)	81.7 (10.9)	83.6 (11.8)	82.5 (11.3)
Age group			
65–79 years	3009 (45.4)	1808 (35.4)	4817 (41.1)
≥ 80 years	3617 (54.6)	3298 (64.6)	6915 (58.9)
Male	2999 (45.3)	2265 (44.4)	5264 (44.9)
No schooling	3857 (58.2)	3052 (59.8)	6909 (58.9)
White-collar workers	1536 (23.2)	951 (18.6)	2487 (21.2)
Urban residents	2695 (40.7)	1783 (34.9)	4478 (38.2)
Married	2771 (41.8)	2150 (42.1)	4921 (41.9)
Living with family members	5509 (83.1)	4235 (82.9)	9744 (83.1)
Smoking status			
Never	4213 (63.6)	3373 (66.1)	7586 (64.7)
Past	963 (14.5)	697 (13.7)	1660 (14.1)
Current	1437 (21.7)	1033 (20.2)	2470 (21.1)
Current alcohol consumption	1549 (23.4)	1011 (19.8)	2560 (21.8)
Currently exercises	2403 (36.3)	1555 (30.5)	3958 (33.7)
Hypertension	1003 (15.1)	1043 (20.4)	2046 (17.4)
Diabetes	114 (1.7)	137 (2.7)	251 (2.1)
Heart disease	558 (8.4)	408 (8.0)	966 (8.2)
Cerebrovascular disease	272 (4.1)	253 (5.0)	525 (4.5)
Respiratory disease	794 (12.0)	464 (9.1)	1258 (10.7)
Body mass index			
Underweight (< 18.5)	2683 (40.5)	1733 (33.9)	4416 (37.6)
Normal (18.5–23.9)	3017 (45.5)	2379 (46.6)	5396 (46.0)
Overweight (24–27.9)	634 (9.6)	622 (12.2)	1256 (10.7)
Obese (≥ 28)	204 (3.1)	245 (4.8)	449 (3.8)
Psychological distress			
1st quartile	1205 (18.2)	884 (17.3)	2089 (17.8)
2st quartile	1870 (28.2)	1419 (27.8)	3289 (28.0)
3st quartile	1804 (27.2)	1295 (25.4)	3099 (26.4)
4st quartile	1358 (20.5)	985 (19.3)	2343 (20.0)
Baseline MMSE score, points^†^	27 [23, 29]	27 [21, 29]	27 [22, 29]
Baseline cognitive impairment^‡^	1740 (26.3)	1680 (32.9)	3420 (29.2)
Final MMSE score, points^†,§^	26 [19, 29]	26 [18, 29]	26 [19, 29]
Final cognitive impairment^‡^	2414 (36.4)	1979 (38.8)	4393 (37.4)
Rate of change in MMSE score, points/year^†,¶^	0.3 [− 0.3, 1.7]	0.0 [− 0.7, 1.4]	0.0 [− 0.6, 1.6]
Cognitive decline^#^			
Improvement	2197 (33.2)	1909 (37.4)	4106 (35.0)
Stable	1094 (16.5)	790 (15.5)	1884 (16.1)
Slow decline	1631 (24.6)	1196 (23.4)	2827 (24.1)
Rapid decline	1704 (25.7)	1211 (23.7)	2915 (24.8)
Vital status			
Survived	4144 (62.5)	3335 (65.3)	7479 (63.7)
Deceased	2482 (37.5)	1771 (34.7)	4253 (36.3)
Duration of follow-up, years^†^			
Survived	3.17 [3.08, 3.25]	2.75 [2.50, 2.83]	3.08 [2.75, 3.17]
Deceased	1.58 [1.00, 2.33]	1.33 [0.75, 2.00]	1.50 [0.92, 2.25]

*Abbreviation*: *MMSE* Mini-Mental State Examination

Note: Missing data: 34 for schooling, 27 for occupation, 16 for smoking, 8 for drinking, 10 for exercising, 346 for hypertension, 342 for diabetes, 328 for heart disease, 304 for cerebrovascular disease, 264 for respiratory disease, 215 for BMI, 912 for symptom of psychological distress

^†^Data are expressed as the median [25th percentile, 75th percentile]

^‡^Cognitive impairment: MMSE score < 24 points

^§^MMSE re-examined at 3 years after baseline measurement

^¶^Rate of change in MMSE score = (baseline MMSE score − final MMSE score)/the interval between two examinations (years)

^#^Cognitive decline was defined according to the rate of change in MMSE score. Improvement—rate of change in MMSE score less than zero; stable—rate of change in MMSE score equal to zero; slow decline—rate of change in MMSE score greater than zero but equal to or less than the median of the rate of change showing decline; rapid decline—rate of change in MMSE score greater than the median of the rate of change showing decline

### Association of cognitive decline with mortality

**Fig. 2:**
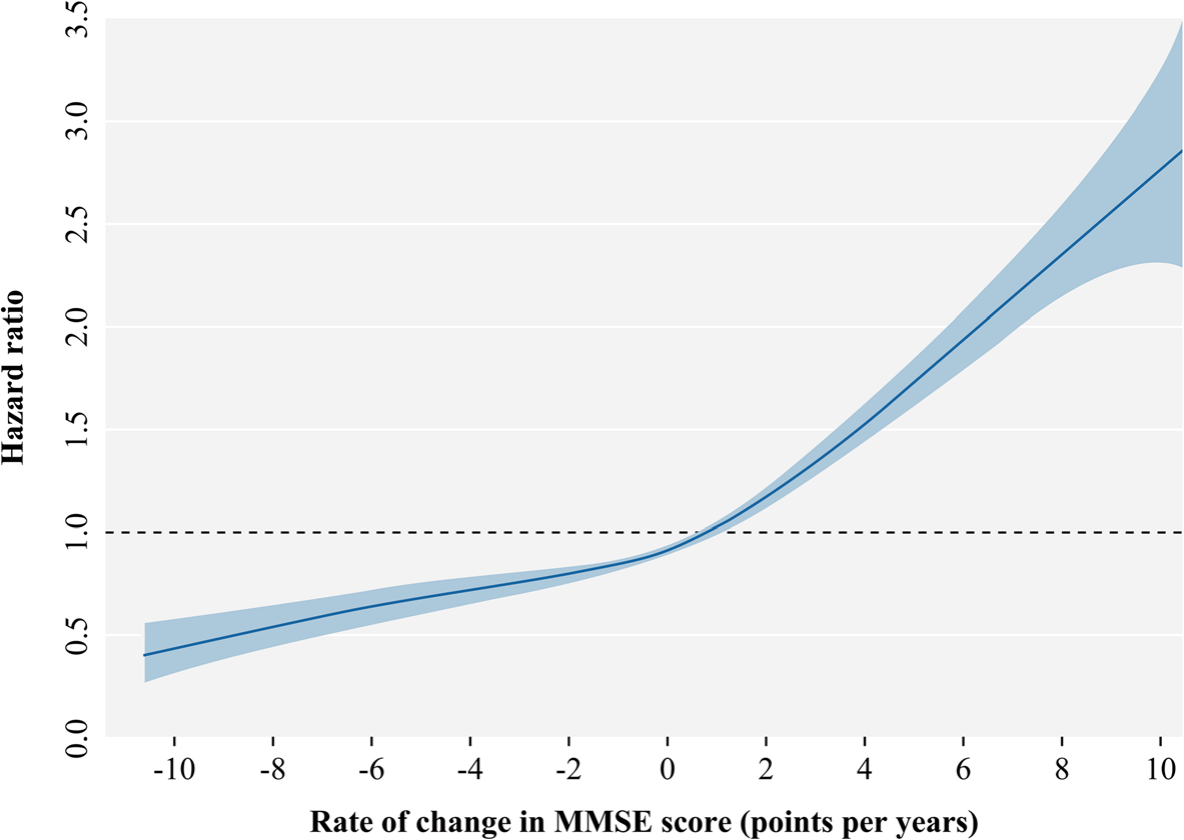
Association of the rate of change in Mini-Mental State Examination (MMSE) score with mortality (hazard ratios are indicated by solid lines and 95% confidence intervals by shaded areas, reference point is the lowest value of rate of change in MMSE score, with knots placed at 5th, 35th, 65th and 95th percentiles), after adjusting for baseline cognitive function, age, sex, cohort, education, occupation, type of residence, marital status, co-residence, smoking, alcohol intake, exercise, hypertension, diabetes, heart disease, cerebrovascular disease, respiratory disease, body mass index and psychological distress

**Fig. 3:**
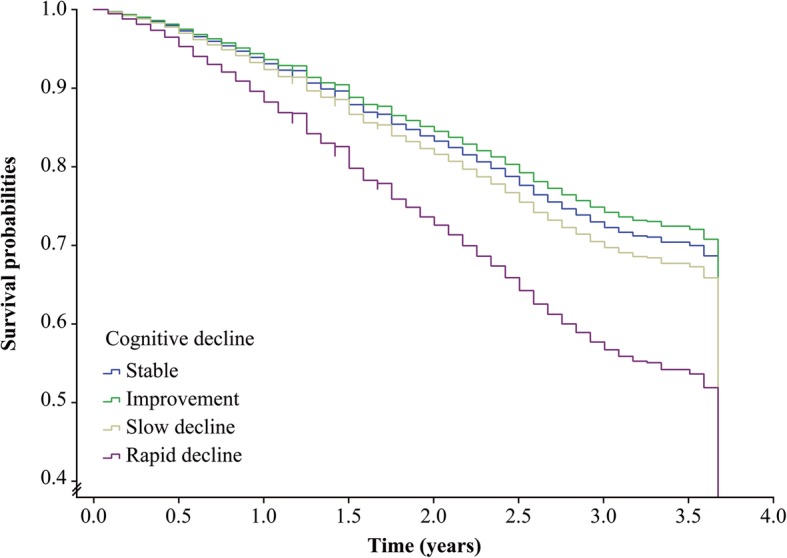
Survival probabilities by the level of cognitive decline after adjusting for baseline cognitive function, age, sex, cohort, education, occupation, type of residence, marital status, co-residence, smoking, alcohol intake, exercise, hypertension, diabetes, heart disease, cerebrovascular disease, respiratory disease, body mass index and psychological distress

Table 2 shows the association of cognitive decline with mortality. A multivariable-adjusted model showed that participants with a one more point reduction in MMSE score per year had an 11% higher risk of death (HR = 1.11, 95%CI 1.10–1.12). We observed a positive and monotonic association between the rate of change in MMSE score and mortality: the faster the cognitive decline, the higher the risk of death (*P* for trend < 0.0001, Fig. 2). Compared to those with stable cognitive function, participants with rapid cognitive decline had 75% higher mortality (HR = 1.75, 95%CI 1.57–1.95). Those with slow cognitive decline and cognitive improvement had a similar risk of death as those with stable cognitive function (Table 2 and Fig. 3).

**Table 2:** The association of 3-year cognitive decline with subsequent 3-year mortality among 11,732 community-dwelling Chinese older people

Model	Cognitive decline HR (95%CI)					
	Continuous	Categorical*				
	Rate of change in MMSE score^†^	Improvement	Stable	Slow decline	Rapid decline	*P* for trend
Model 1^‡^	1.06 (1.05–1.07)	1.01 (0.91–1.13)	Reference	1.00 (0.89–1.12)	1.56 (1.40–1.73)	< 0.0001
Model 2^§^	1.08 (1.07–1.09)	0.99 (0.81–1.10)	Reference	1.05 (0.94–1.18)	1.65 (1.48–1.84)	< 0.0001
Model 3^¶^	1.11 (1.10–1.12)	0.92 (0.82–1.02)	Reference	1.11 (0.99–1.25)	1.75 (1.57–1.95)	< 0.0001

*Abbreviations*: *HR* hazard ratio, *CI* confidence interval, *MMSE* Mini-Mental State Examination

Note: Number of deaths/person years for each category of cognitive decline: 1340/10205, 461/4192, 814/7309 and 1638/6208

*Improvement—rate of change in MMSE score less than zero; stable—rate of change in MMSE score equal to zero; slow decline—rate of change in MMSE score greater than zero but equal to or less than the median of the rate of change showing decline; rapid decline—rate of change in MMSE score greater than the median of the rate of change showing decline

^†^Rate of change in MMSE score = (baseline MMSE score − final MMSE score)/the interval between two examinations (years)

^‡^Age and sex were adjusted

^§^Cohort, education, occupation, type of residence, marital status, co-residence, smoking, alcohol intake, exercise, hypertension, diabetes, heart disease, cerebrovascular disease, respiratory disease, body mass index and psychological distress were additionally adjusted

^¶^Baseline MMSE score was additionally adjusted

### Subgroup analysis

**Fig. 4:**
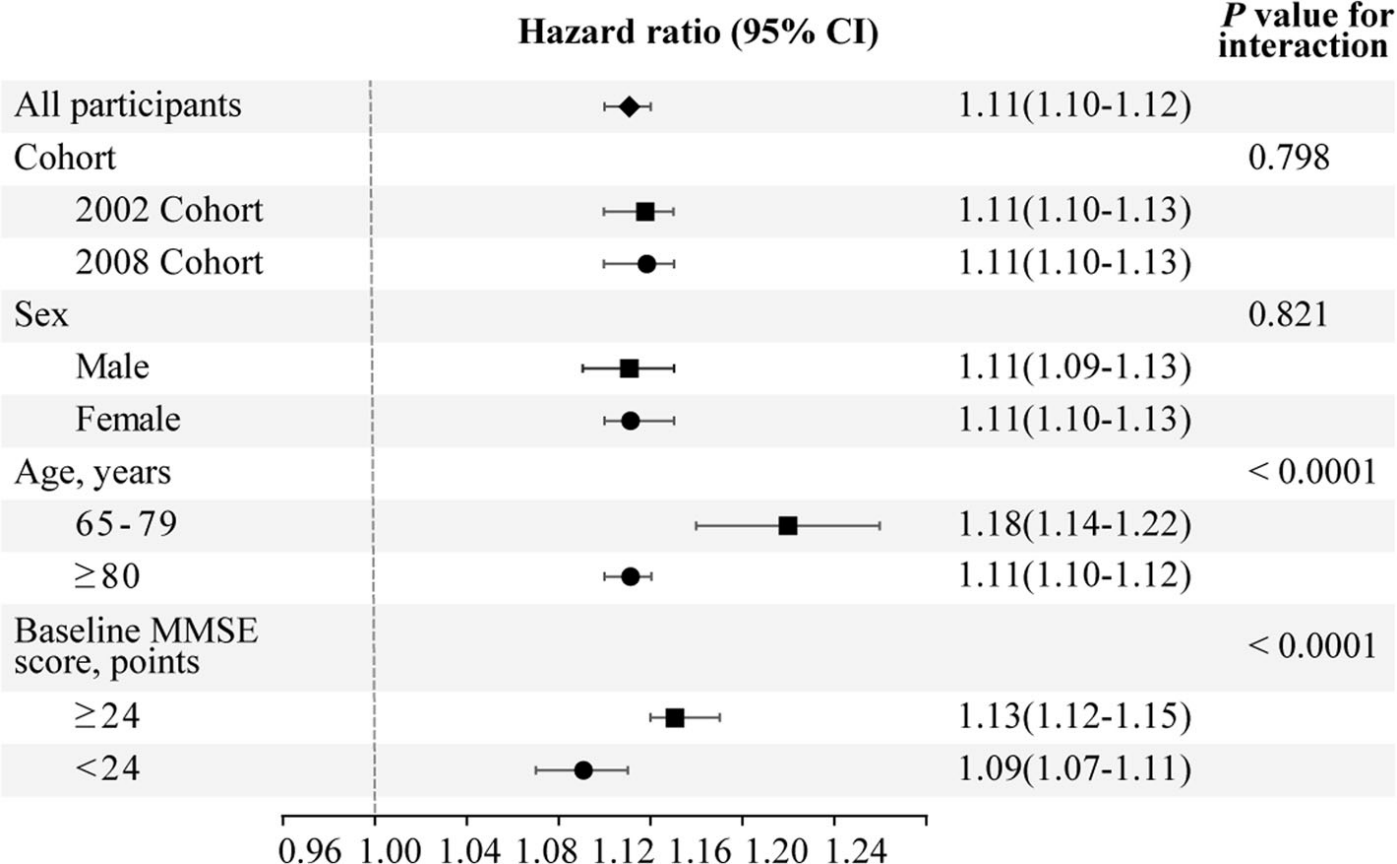
Hazard ratios and 95% confidence intervals (CIs) for the association between the rate of change in the Mini-Mental State Examination (MMSE) score and mortality by cohort, sex, age and baseline cognitive function, adjusting for baseline cognitive function, age, sex, cohort, education, occupation, type of residence, marital status, co-residence, smoking, alcohol intake, exercise, hypertension, diabetes, heart disease, cerebrovascular disease, respiratory disease, body mass index and psychological distress

**Fig. 5:**
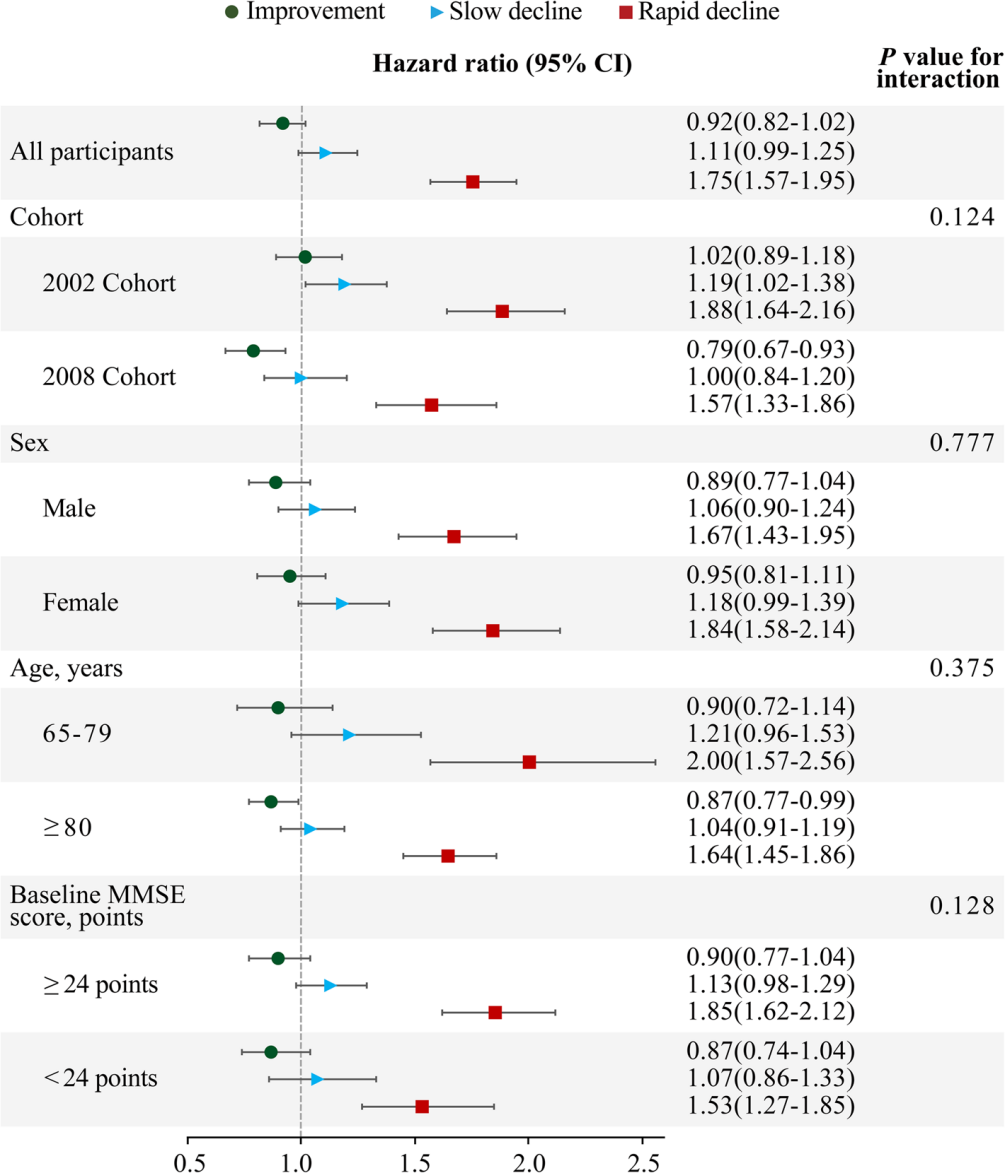
Hazard ratios and 95% confidence intervals (CIs) for the association between cognitive decline and mortality, with stable cognitive function as a reference group, by cohort, sex, age and baseline cognitive function, adjusting for baseline cognitive function, age, sex, cohort, education, occupation, type of residence, marital status, co-residence, smoking, alcohol intake, exercise, hypertension, diabetes, heart disease, cerebrovascular disease, respiratory disease, body mass index and psychological distress

The association between the rate of change in MMSE score and mortality among those aged 65–79 years was stronger than that among those aged 80 years and over and among those with baseline MMSE scores ≥ 24 points compared to those with MMSE scores < 24 points but did not differ by cohort and sex (Fig. 4). The association of categorical cognitive decline with mortality did not differ by cohort, sex, age or baseline cognitive function, although it appeared stronger in the 2002 cohort, among women, among relatively younger Chinese older people and among those with baseline MMSE scores ≥ 24 points (Fig. 5).

### Sensitivity analysis

Our findings remained robust when we treated participants lost to follow-up as censored at the third survey or excluded participants who died in the first year of follow-up (Table 3).

**Table 3:** Sensitivity analyses for the association of 3-year cognitive decline with subsequent 3-year mortality

	Hazard ratio (95%CI)	
	Model 1* (*n* = 13,594)	Model 2^†^ (*n* = 10,549)
Rate of change in MMSE score^‡^	1.11 (1.10–1.12)	1.10 (1.09–1.11)
Cognitive decline^§^		
Improvement	0.90 (0.81–1.00)	0.98 (0.86–1.12)
Stable	Reference	Reference
Slow decline	1.09 (0.97–1.23)	1.17 (1.02–1.35)
Rapid decline	1.68 (1.51–1.87)	1.74 (1.53–1.98)

*Abbreviations*: *MMSE* Mini-Mental State Examination, *CI* confidence interval

In models 1 and 2, we adjusted for baseline MMSE score, age, sex, cohort, education, occupation, type of residence, marital status, co-residence, smoking, drinking, exercise, hypertension, diabetes, heart disease, cerebrovascular disease, respiratory disease, body mass index and psychological distress

*Model 1: participants lost to follow-up were treated as censored at the end of the study

^†^Model 2: exclusion of deaths that occurred in the first year of follow-up

^‡^Rate of change in MMSE score = (baseline MMSE score − final MMSE score)/the interval between two examinations (years)

^§^Cognitive decline was defined according to the rate of change in MMSE score. Improvement—rate of change in MMSE score less than zero; stable—rate of change in MMSE score equal to zero; slow decline—rate of change in MMSE score greater than zero but equal to or less than the median of the rate of change showing decline; rapid decline—rate of change in MMSE score greater than the median of the rate of change showing decline

## Discussion

In this large prospective and community-based study, after adjusting for baseline cognitive function and other confounders, we found that the rate of change in an individual’s MMSE score over 3 years was monotonically and positively associated with mortality. Participants with rapid cognitive decline had higher mortality during the subsequent 3 years. The association between cognitive decline and mortality was stronger among relatively younger Chinese older people (aged 65–79 versus ≥ 80+ years) and those with normal cognitive function at baseline (MMSE score ≥ 24 versus < 24 points) but did not differ by cohort and sex.

In line with most previous studies [5, 13–18], the findings from our data suggest that cognitive decline is related to mortality independent of initial cognitive function. Our results suggest that rapid decline in cognitive function may be a sign of the approach of life’s end. Considering the effect of cognitive training interventions on cognitive function [32], it is possible that maintaining cognitive function would be beneficial for survival in older adults, even for those already experiencing lower cognitive function. It has been reported that vascular risk factors, vascular diseases and psychological well-being are associated with cognitive decline [33–36]. The associations of cognitive decline with mortality in our study did not substantially change after adding comorbid diseases to the models (Table 2), which is similar to previous studies [14–16]. This evidence suggests that the association between cognitive decline and mortality might not be attributed to underlying diseases. Whether cognitive decline is a marker of biological ageing or plays a causative role in longevity or whether some possible factors simultaneously contribute to both cognitive decline and mortality deserves further investigation.

The association between cognitive decline and mortality was not substantially different in the 2008 cohort compared to the 2002 cohort. Similar to previous studies [7, 9], we found no evidence for a differing cognitive decline-mortality association between men and women. Consistent with previous studies [5, 16, 19], we observed that age influenced the association of cognitive decline with mortality. That is, relatively younger Chinese older people (65–79 versus 80+ years) had a higher risk of death related to cognitive decline. It has been suggested that cognitive decline among relatively younger older people may reflect underlying brain disease or some potential processes that are associated with increased risk of death [5, 14]. Moreover, the smaller association of cognitive decline and mortality among individuals aged 80 years and over may be partially explained by survival bias. We also found that those with baseline MMSE scores ≥ 24 points had higher mortality related to cognitive decline compared to those with MMSE scores < 24 points, which suggests that those with normal cognitive function would probably benefit more from cognitive function preservation than those already cognitively impaired. In addition, owing to the lower sensitivity of MMSE for mild impairment [37], there might be some overlap between the group with MMSE scores ≥ 24 points and those with MMSE scores < 24 points. Therefore, the difference in the association of cognitive decline and mortality between individuals with normal cognitive function and those with cognitive impairment at baseline might be attenuated in our study. Overall, our findings indicate that preventing cognitive decline as early as possible would potentially extend a patient’s lifespan.

One of our study’s limitations was that only participants who completed two cognitive examinations (for assessing cognitive decline) were included in the present analyses. Those who only completed the baseline survey had lower initial cognitive function (data not shown). The risk of death related to cognitive decline was slightly weaker among those with cognitive impairment; therefore, the association between cognitive decline and mortality in our study sample might be slightly higher than that in the whole population. In addition, practice effects have been found to persist over periods up to 7 years [38]. The average interval of two MMSE examinations was 3 years in the current study, and the practice effects would have underestimated decline and therefore its impact. Moreover, it was reported that the rate of terminal cognitive decline can be highly variable but is likely to accelerate approximately 3 years before death [39]. Our findings have limited generalizability to the association of long-term cognitive decline with mortality. Furthermore, some potential covariates, either unmeasured (e.g. medical treatment, inflammation, diet) [4, 7] or unknown, might confound the association between cognitive decline and mortality. On the other hand, we accounted for a set of common confounders, and the results were robust. Residual confounding was another potential concern.

The strengths of this study included the large sample size, its prospective nature and a community-based representative sample. To our knowledge, this is the first study that investigated the relationship between cognitive decline and mortality in a national cohort of Chinese older people. The current study included a large population of Chinese older adults aged 80 years and over, allowing a robust assessment of the association between cognitive decline and mortality among this group. Additionally, we used the same protocol for sampling and data collection in the two cohorts; therefore, they were comparable to some extent (Table 1).

Given that cognitive function in older adults may be modifiable [40], our findings have potential public health significance. The results indicate the practical significance of the detection of cognitive decline with the MMSE or other brief instruments in community settings, especially for relatively young Chinese older people and those with normal cognitive function.

## Conclusions

Cognitive decline, independent of initial cognitive function, was associated with higher mortality. Individuals aged 65–79 years and those with normal cognitive function may have a higher risk of death related to cognitive decline. The association was strong and consistent across two large and successive cohorts. This study provides evidence for monitoring cognitive change in older adults.

## Abbreviations

BMI: Body mass index
CI: Confidence interval
CLHLS: Chinese Longitudinal Healthy Longevity Survey
HR: Hazard ratio
MMSE: Mini-Mental State Examination
